# IPAD: the Integrated Pathway Analysis Database for Systematic Enrichment Analysis

**DOI:** 10.1186/1471-2105-13-S15-S7

**Published:** 2012-09-11

**Authors:** Fan Zhang, Renee Drabier

**Affiliations:** 1 Department of Academic and Institutional Resources and Technology, University of North Texas Health Science Center, Fort Worth, USA; 2 Department of Forensic and Investigative Genetics, University of North Texas Health Science Center, Fort Worth, USA

## Abstract

**Background:**

Next-Generation Sequencing (NGS) technologies and Genome-Wide Association Studies (GWAS) generate millions of reads and hundreds of datasets, and there is an urgent need for a better way to accurately interpret and distill such large amounts of data. Extensive pathway and network analysis allow for the discovery of highly significant pathways from a set of disease vs. healthy samples in the NGS and GWAS. Knowledge of activation of these processes will lead to elucidation of the complex biological pathways affected by drug treatment, to patient stratification studies of new and existing drug treatments, and to understanding the underlying anti-cancer drug effects. There are approximately 141 biological human pathway resources as of Jan 2012 according to the Pathguide database. However, most currently available resources do not contain disease, drug or organ specificity information such as disease-pathway, drug-pathway, and organ-pathway associations. Systematically integrating pathway, disease, drug and organ specificity together becomes increasingly crucial for understanding the interrelationships between signaling, metabolic and regulatory pathway, drug action, disease susceptibility, and organ specificity from high-throughput omics data (genomics, transcriptomics, proteomics and metabolomics).

**Results:**

We designed the Integrated Pathway Analysis Database for Systematic Enrichment Analysis (IPAD, http://bioinfo.hsc.unt.edu/ipad), defining inter-association between pathway, disease, drug and organ specificity, based on six criteria: 1) comprehensive pathway coverage; 2) gene/protein to pathway/disease/drug/organ association; 3) inter-association between pathway, disease, drug, and organ; 4) multiple and quantitative measurement of enrichment and inter-association; 5) assessment of enrichment and inter-association analysis with the context of the existing biological knowledge and a "gold standard" constructed from reputable and reliable sources; and 6) cross-linking of multiple available data sources.

IPAD is a comprehensive database covering about 22,498 genes, 25,469 proteins, 1956 pathways, 6704 diseases, 5615 drugs, and 52 organs integrated from databases including the BioCarta, KEGG, NCI-Nature curated, Reactome, CTD, PharmGKB, DrugBank, PharmGKB, and HOMER. The database has a web-based user interface that allows users to perform enrichment analysis from genes/proteins/molecules and inter-association analysis from a pathway, disease, drug, and organ.

Moreover, the quality of the database was validated with the context of the existing biological knowledge and a "gold standard" constructed from reputable and reliable sources. Two case studies were also presented to demonstrate: 1) self-validation of enrichment analysis and inter-association analysis on brain-specific markers, and 2) identification of previously undiscovered components by the enrichment analysis from a prostate cancer study.

**Conclusions:**

IPAD is a new resource for analyzing, identifying, and validating pathway, disease, drug, organ specificity and their inter-associations. The statistical method we developed for enrichment and similarity measurement and the two criteria we described for setting the threshold parameters can be extended to other enrichment applications. Enriched pathways, diseases, drugs, organs and their inter-associations can be searched, displayed, and downloaded from our online user interface. The current IPAD database can help users address a wide range of biological pathway related, disease susceptibility related, drug target related and organ specificity related questions in human disease studies.

## Background

With the age of big data approaching [1], bioinformatics for Next-Generation Sequencing (NGS) and Genome-Wide Association Studies (GWAS) will be one of the biggest areas of disruptive innovation in life science tools over the next few years [2]. Next-Generation Sequencing technologies and Genome-Wide Association Studies generate millions of reads and hundreds of datasets, and there is an urgent need for a better way to accurately interpret and distill such large amounts of data. The use of large scale gene expression analysis has been proven to be useful in identifying differentially expressed genes to classify and predict various disease subtypes. However, it is often difficult to assign biological significance to a large number of genes that are implicated. This problem persists even when users are able to reduce the number of differentially expressed genes substantially via hierarchical clustering methods.

As more information is revealed through large-scale "omics" techniques, it is becoming increasingly apparent that genes do not function alone but through complex biological pathways. Unraveling these intricate pathways is essential to understanding biological mechanisms, disease states, and the function of drugs that transform them. Extensive pathway and network analysis allow for the discovery of highly significant pathways from a set of disease vs. healthy samples in the NGS and GWAS. Knowledge of activation of these processes will lead to elucidation of the complex biological pathways affected by drug treatment, to patient stratification studies of new and existing drug treatments, and to understanding the underlying anti-cancer drug effects.

Pathway databases serve as repositories of current knowledge on cell signaling, enzymatic reaction, and genetic regulation. There are more than 300 pathway repositories listed in Pathguide resource http://www.pathguide.org[3], from which over 141 are specialized on reactions in human as of Jan 2012, for example, BioCarta http://www.biocarta.com[4], KEGG http://www.genome.jp/kegg/[5], NCI-Nature curated http://pid.nci.nih.gov/PID/index.shtml[6], Reactome http://www.reactome.org[7], and Wikipathways http://www.wikipathways.org/[8]. However, these resources have several limitations. First, most currently available resources do not contain disease, drug or organ specificity information such as disease-pathway, drug-pathway, and organ-pathway associations. Next, these resources have been developed with variable degrees of data coverage, quality, and utility [9]. In addition, only half of them provide pathways and reactions in computer-readable formats needed for automatic retrieval and processing [10]. Lastly, many pathway databases are in distinct formats [11].

**Figure 1:**
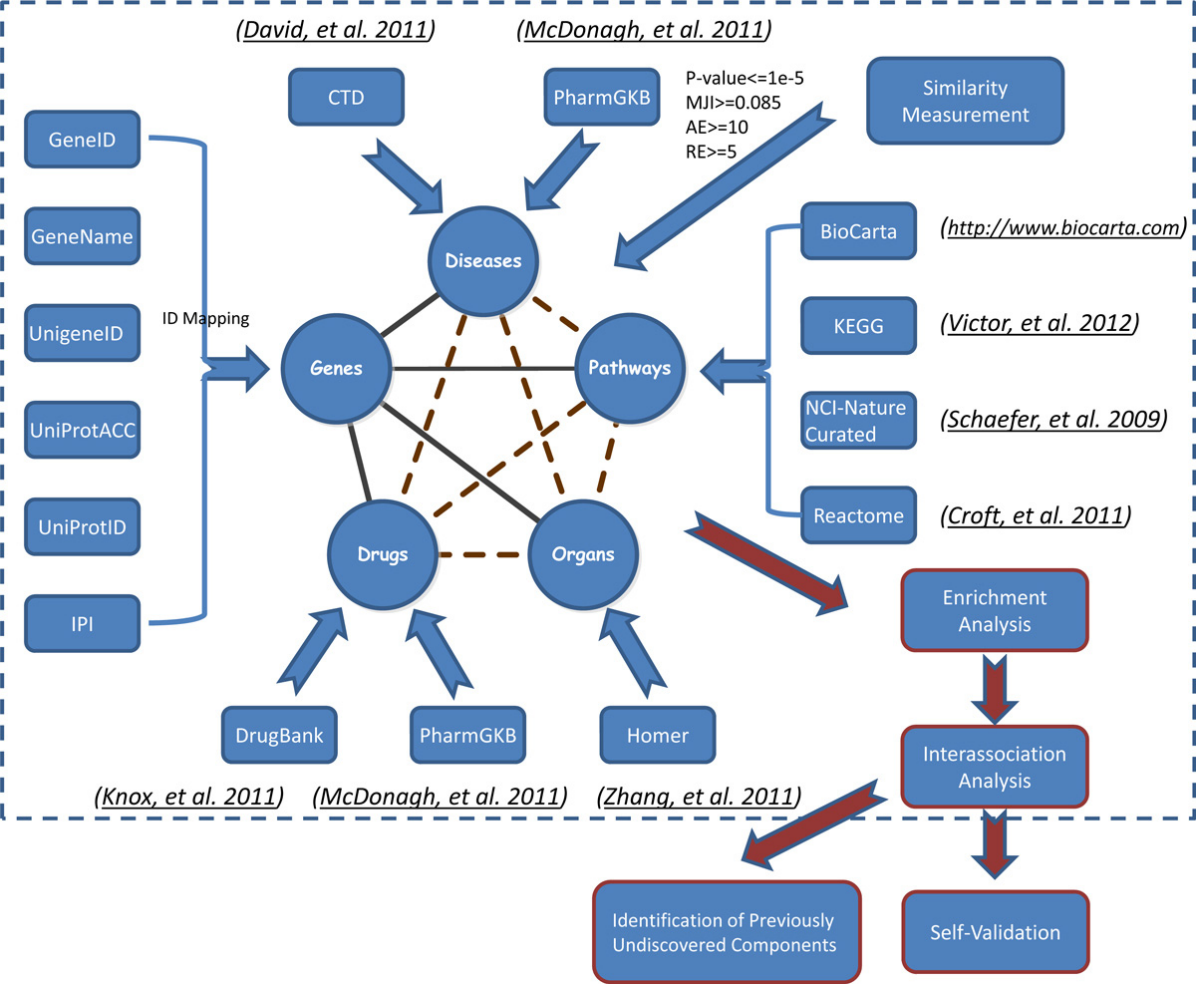
**Data Integration Process**. The whole data integration process was divided into three steps: 1) associations of molecule-pathway, molecule-disease, molecule-drug, molecule-organ; 2) inter-association analysis between pathway, disease, drug and organ; and 3) enrichment analysis and inter-association analysis: 3a) self-validation and 3b) identification of previously undiscovered components by the enrichment analysis.

Systematic collection of pathway information not only in the form of pathway databases but also including inter-association between pathway, disease, drug, and organ specificity is crucial, because 1) it provides a bridge between pathway, disease, drug and organ, and 2) this bridge can not only capture relevant biological pathways but also provide disease, drug target, and organ specificity information. For "inter-association", we refer to a biological connection between two or more biological components on basis of intermediary genes (dotted lines in Figure 1).

A component is a biomedical concept such as pathway, disease, drug and organ (nodes in Figure 1). Some pilot studies about this kind of connections have been done in the past. For example, Li et al. investigated disease relationships based on their shared pathways [12]. First, they extracted disease associated genes by literature mining. Then, they connected diseases to biological pathways through overlapping genes. Lastly, they built a disease network by connecting diseases sharing common pathways. Smith et al. combined pathway analysis and drug analysis to identify common biological pathways and drug targets across multiple respiratory viruses based on human host gene expression analysis. Their study suggested that multiple and diverse respiratory viruses invoked several common host response pathways [13]. One study found that disease candidate genes were functionally related in the form of protein complexes or biological pathways and complex disease ensued from the malfunction of one or a few specific signaling pathways [14]. Another study aimed to explore complex relationships among diseases, drugs, genes, and target proteins altogether [15] and found that mapping the polypharmacology network onto the human disease-gene network revealed not only that drugs commonly acted on multiple targets but also that drug targets were often involved with multiple diseases. Berger and Iyengar also discussed how analysis of biological networks had contributed to the genesis of systems pharmacology and how these studies had improved global understanding of drug targets [16]. They described that an emerging area of pharmacology, systems pharmacology, which utilizes biological network analysis of drug action as one of its approaches, is becoming increasingly important in: providing new approaches for drug discovery for complex diseases; considering drug actions and side effects in the context of the regulatory networks within which the drug targets and disease gene products function; understanding the relationships between drug action and disease susceptibility genes; and increasing knowledge of the mechanisms underlying the multiple actions of drugs [16].

Therefore we created the Integrated Pathway Analysis Database for Systematic Enrichment Analysis (IPAD) for users to query information about genes, diseases, drugs, organ specificity, and signaling and metabolic pathways. First, we integrated data from four kinds of sources: 1) pathway databases from BioCarta [4], KEGG [5], NCI-Nature curated [6], and Reactome [7], 2) disease databases from CTD http://ctdbase.org/[17] and PharmGKB http://www.pharmgkb.org[18], 3) drug databases from DrugBank httP://www.drugbank.ca[19] and PharmGKB [18], and 4) organ-specific genes/proteins from HOMER http://discern.uits.iu.edu:8340/Homer/index.html[20]. Next, we created inter-association between pathway, disease, drug, and organ specificity. Then, we built a web interface for users to perform 1) enrichment analysis from genes/proteins/molecules, and 2) inter-association analysis from a pathway, disease, drug and organ. Lastly, we presented three case studies: 1) breast cancer related markers, 2) brain-specific markers, and 3) prostate cancer model to demonstrate that the IPAD can enable users to analyze enrichment and inter-association between pathway, disease, drug and organ, to discover previously undiscovered pathway, disease, drug and organ, and to validate the enrichments.

The Integrated Pathway Analysis Database for Systematic Enrichment Analysis (IPAD), located at http://bioinfo.hsc.unt.edu/ipad is a comprehensive database covering about 22,498 genes, 25,469 proteins, 1956 pathways, 6704 diseases, 5615 drugs, and 52 organs integrated from databases including the BioCarta [4], KEGG [5], NCI-Nature curated [6], Reactome [7], CTD [17], PharmGKB [18], DrugBank [19], PharmGKB [18], and HOMER [20].

It is the first comprehensive database that can be used to analyze, discover, and validate enrichment and inter-association between pathway, disease, drug and organ. The inter-associations allow further identification of enriched pathways, diseases, drugs and organs. The quality of the database is validated on a "gold standard" constructed from reputable and reliable sources. The ability to choose multiple quantitative parameters (p-value, Absolute Enrichment Value (AE), Relative Enrichment Value (RE), and Mean Jaccard Index (MJI)) provides us with powerful statistics and computation to accurately calculate enrichment and inter-association. And the cross-linking of multiple data sources enables subsequent validation.

## Results

### Database content statistics

By integrating pathway, disease, drug, and organ specificity databases including BioCarta [4], KEGG [5], NCI-Nature curated [6], Reactome [7], CTD [17], PharmGKB [18], DrugBank [19], and Homer [20], we have developed IPAD, the Integrated Pathway Analysis Database for systematic enrichment analysis. As of the current release (May 2012), IPAD contains 25,469 proteins (IPI IDs), 22,498 genes (gene IDs), 1956 pathways covering 11663 genes, 6,704 diseases covering 17925 genes, 5,615 drugs covering 3735 genes, and 52 organs covering 5599 genes (Table 1). A comparison of pathways in IPAD against several common pathway data sources is shown in Table 2.

**Table 1:** Current Statistics of Database

Total Number	Count
genes	22,498 GeneIDs
proteins	25,469 UniProtIDs
Pathways	1956 (BioCarta:310,KEGG:247, NCI-Nature curated:222, Reactome:1177)
Molecules in Pathway	11663
Diseases	6704(CTD:5892, PharmGKB:812)
Molecules in Disease	17925
Drugs	5615(DrugBank:4604, PharmGKB:1011)
Molecules in Drug	3735
Organs	52
Molecules in Organ	5599

**Table 2:** A Comparison of Human Pathways in IPAD against Several Common Pathway Data Sources

	**BioCarta**[4]	**KEGG**[5]	**NCI-Nature curated**[6]	**Reactome**[7]	IPAD
Pathway coverage	310	247	222	1177	1956
Molecule coverage	1372	9238	2561	5668	11663
Last Updated	2010	Mar 2012	July 2010	Jan 2011	Mar 2012
Curation Type	Manual	Manual	Manual	Manual	Integrated
Disease Association	No	Yes	No	No	Yes
Drug Association	No	Yes	No	No	Yes
Organ Specificity Association	No	No	No	No	Yes
Inter-associations Quantitative	No	No	No	No	Yes
Enrichment Score Quantitative	No	No	No	No	Yes
Similarity	No	No	No	No	Yes

### P-value distribution of inter-association

**Figure 2:**
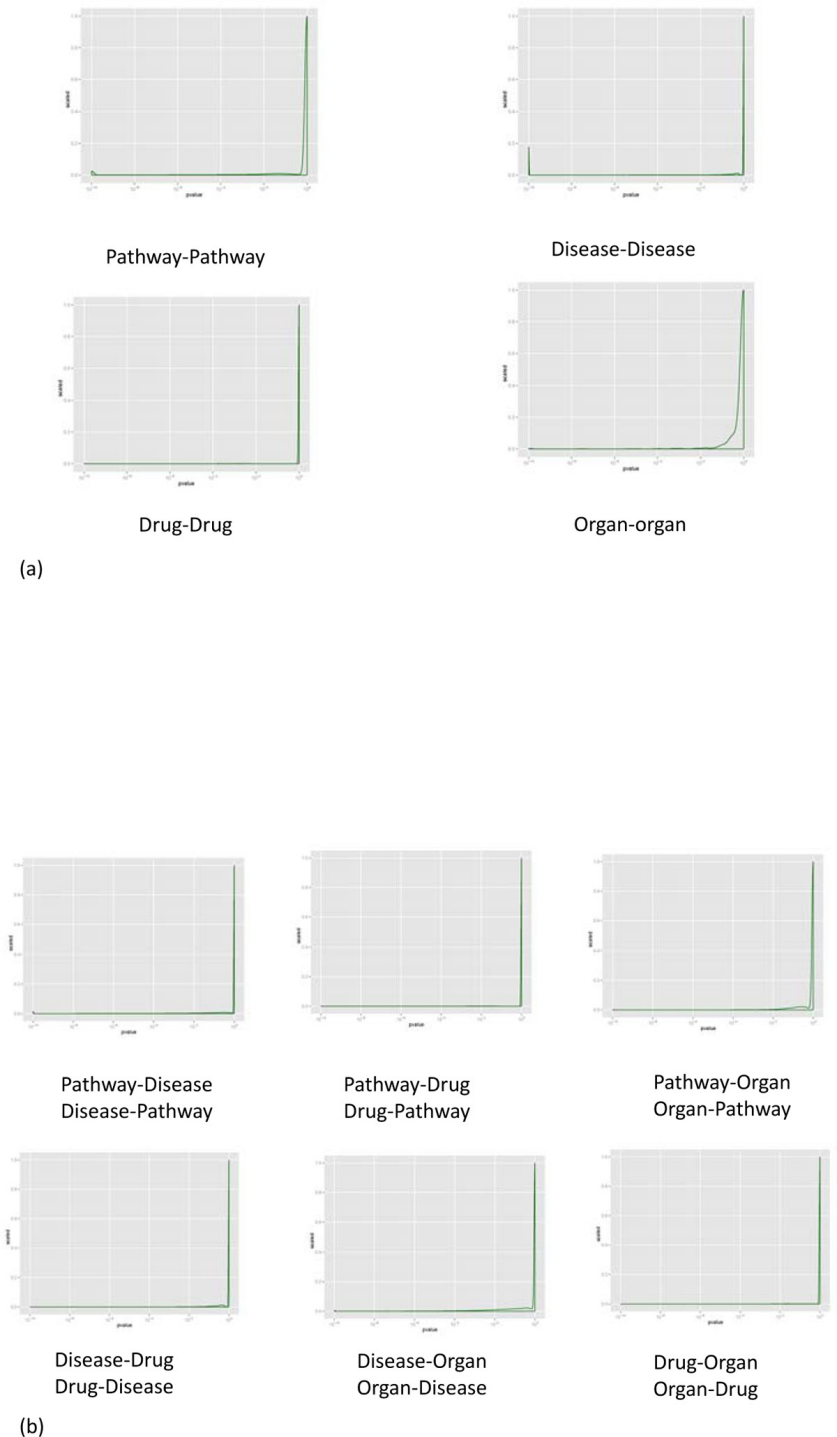
**p-value Distribution of Inter-association**. The y-axis is the scaled density of p-value which is calculated by Fisher Exact test. (a) p-value Distribution of Inter-association between pathway-pathway, disease-disease, drug-drug, and organ-organ. (b) p-value Distribution of Inter-association between pathway, disease, drug, and organ.

We performed statistical testing using p-value described in the method section to describe the inter-association between pathway, disease, drug and organ in IPAD (Figure 2a and Figure 2b). Although the majority of associations are not significant (p-value close to 1), there are still some which are significant (p-value ≤ 10^-5^). Component similarity can also be measured by Absolute Expression Value (AE), Relative Expression Value (RE) and Mean Jaccard Index (MJI). The four measurements (p-value, AE, RE, MJI) can complement each other and compensate for the weaknesses inherent in each alone to create better criteria for enrichment analysis.

**Figure 3:**
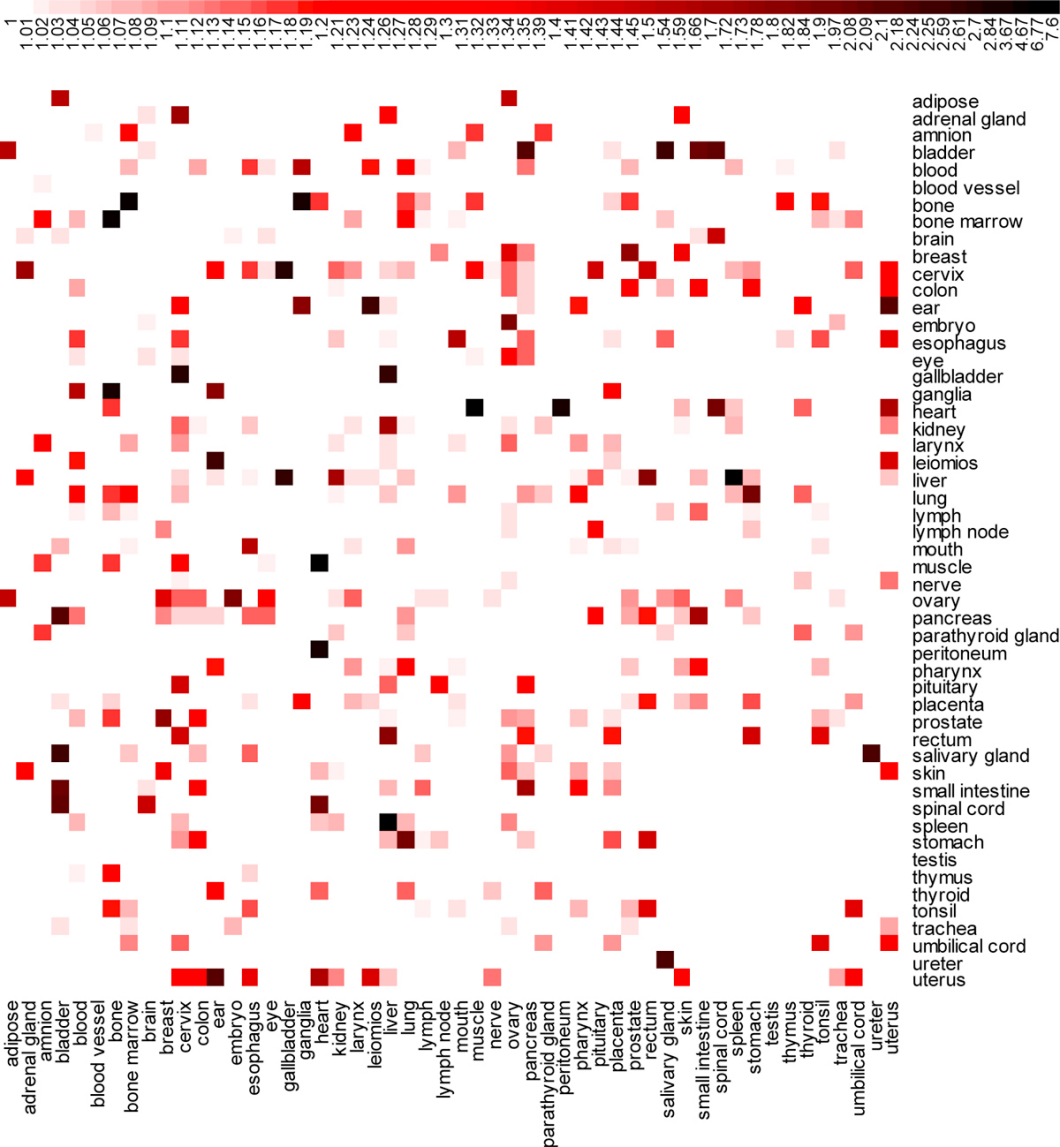
**Heatmap of Inter-associations between 52 Organs**. x-axis and y-axis are both 52 organs. The degrees of redness and blackness in each cell represent increase of association between organs. The legend above the heatmap indicates the range of association between organs. The association between organs is expressed by 1 minus log10 of p-value. It is nonlinear color scale from white to red to black, correspondingly, indicating the value of 1-log10(p-value) scales from 1 to 7.6.

The inter-association between the 52 organs in Figure 3 shows that the heart and muscle have strongest association with a smallest p-value:2.51e-7 (1-log10p-value = 7.6) and 14 genes in common. The other strong associations occur between spleen and liver (20 genes in common, p-value = 1.69e-6, and 1-log10p-value = 6.77), and bone marrow and bone (7 genes in common, p-value = 2.15e-4, and 1-log10p-value = 4.67).

### General online features

**Figure 4:**
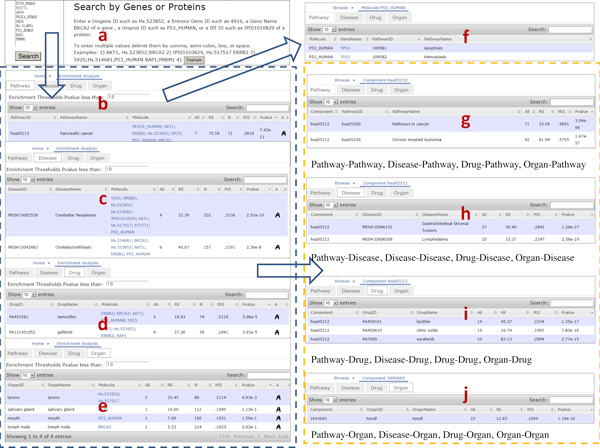
**Web Interface Structure**. a) Query by genes or proteins. For example, UniGene IDs, Entrez gene IDs, gene names, UniProt IDs, UniProt Accessions or IPI IDs are all supported. To enter multiple values, delimit them by comma, semi-colon, line or space. b,c,d,e) search result. In the enrichment analysis table, it shows Pathway ID (Disease ID, Drug ID, Organ ID), Pathway Name (Disease Name, Drug Name, Organ Name), Molecule, significance (AE, RE, N, MJI, p-value), and inter-association. For each enriched component, users can further browse the inter-association between its molecule and pathway, disease, drug and organ by clicking on the link in the column of molecule, and its inter-association between pathway, disease, drug and organ by clicking on the inter-association icon in the last column. f) molecule inter-association. It shows molecule, Gene Symbol, Pathway ID (Disease ID, Drug ID, Organ ID), and Pathway Name (Disease Name, Drug Name, Organ Name). g,h,i,j) inter-association between pathway, disease, drug and organ. It shows associations between Pathway-Pathway, Pathway-Disease, Pathway-Drug, Pathway-Organ, Disease-Pathway, Disease-Disease, Disease-Drug, Disease-Organ, Drug-Pathway, Drug-Disease, Drug-Drug, Drug-Organ, Organ-Pathway, Organ-Disease, Organ-Drug, Organ-Organ, and their significance (AE, RE, MJI, p-value).

In Figure 4, we show the user interfaces of the web-based online version of IPAD. It supports standard and powerful search options that allow users to specify a list of genes/proteins as the query input. Some interesting features of IPAD include the ability to browse for pathway, disease, drug, and organ with tabs in one page, search by keyword in the Search Box over the table, and set the p-value cutoff in the enrichment threshold box to select enriched pathway sets, disease sets, drug sets and organ sets.

In response to the query input, IPAD can retrieve a list of related components (pathways, diseases, drugs, and organs) in a highly flexible table, with which users can further explore details about inter-association between the components. For example, users can browse the inter-association between each component's molecule and pathway, disease, drug and organ by clicking on the link in the column of molecule, and look through the component-related inter-association between pathway, disease, drug and organ by clicking on the inter-association icon in the last column. There are totally sixteen types of inter-associations between pathway, disease, drug and organ in IPAD: Pathway-Pathway, Pathway-Disease, Pathway-Drug, Pathway-Organ, Disease-Pathway, Disease-Disease, Disease-Drug, Disease-Organ, Drug-Pathway, Drug-Disease, Drug-Drug, Drug-Organ, Organ-Pathway, Organ-Disease, Organ-Drug, and Organ-Organ. User queried inter-association pathway/disease/drug/organ data stored in IPAD can also be freely downloaded as tab-delimited text files using links below each enrichment or inter-association table.

### Assessment of IPAD

Assessing the capabilities of any pathway/disease/drug/organ enrichment analysis in real experiments is a challenge in itself because the full truth of what really occurred between the components and how they are actually inter-associated, if at all, may never be known. In the absence of a "gold standard" - a reference standard against which to establish the performance of the filter, the best alternative is to analyze the results of the enrichment analysis method in the context of the existing biological knowledge [21]. We first used two identified studies to illustrate how well the significant pathways/diseases/drugs/organs identified by the enrichment analysis and inter-association analysis of IPAD fit with the existing biological knowledge. Then we constructed a "gold standard" of 30161 known associations and used it to assess the inter-association analysis of IPAD.

### Assessment of enrichment analysis

The absence of a definitive answer regarding the involvement of a particular pathway/disease/drug/organ in a given condition makes it impossible to calculate exact values for sensitivity, specificity, ROCs, etc. Therefore, we compared the result of IPAD's enrichment analysis and inter-association analysis and tested whether the significant pathways/diseases/drugs/organs fit with the existing biological context. This type of assessment is the current best practice in this area of enrichment analysis [22].

In the first dataset, we assessed the features of IPAD by testing the inter-association between breast cancer markers related pathway, disease, drug and organ. Breast cancer is a cancer that starts in the tissues of the breast. We first downloaded the 15 breast cancer related genes from the Cancer Gene Census [23]: AKT1, BAP1, BRCA2, CCND1, CDH1, EP300, ERBB2, ETV6, GATA3, MAP2K4, NTRK3, PBRM1, PIK3CA, RB1, and TP53. The top 5 associated drugs (p-value ≤ 9.9 × 10^-3^, *AE *≥ 2.57, *RE *≥ 13.51 and *MJI *≥ 0.154; PA451581 tamoxifen, PA131301952 gefitinib, PA152241907 lapatinib, PA449509 estrogens, and PA449383 docetaxel) we identified using IPAD are all reportedly linked to breast cancer by previously published papers (Table 3). For example, most women with estrogen-sensitive breast cancer benefit from the drug tamoxifen [24]. This drug blocks the effects of estrogen, which can help breast cancer cells survive and grow. Green et al. tested whether Gefinitib as an orally active selective EGFR inhibitor might benefit advanced breast cancer (ABC) patients either with acquired hormone resistance or with hormone receptor (HR)-negative tumors. They concluded that at a dose of 500 mg/day, gefitinib monotherapy resulted in a low Clinical Benefit Rate (CBR) and no tumor response was identified [25]. Lapatinib is used as a treatment for treatment-naive women with breast cancer, ER+/EGFR+/HER2+ breast cancer patients (now often called "triple positive") and patients who have HER2-positive advanced breast cancer that has progressed after previous treatment with other chemotherapeutic agents, such as anthracycline, taxane-derived drugs, or trastuzumab [26]. Estrogen is a hormone that is necessary for the normal development and growth of the breasts and organs important for childbearing. For example, several weeks after a study suggested that women who take estrogen-only hormone replacement to treat menopause symptoms may be at lower risk for developing breast cancer, another, much-larger study found that when used for longer than 10 years, estrogen-only regimens actually raise a woman's long-term risk for breast cancer [27]. Docetaxel (given with doxorubicin and cyclophosphamide) is recommended as a possible adjuvant treatment for women with early node-positive breast cancer. For example, Martin et al. compared docetaxel plus doxorubicin and cyclophosphamide (TAC) with fluorouracil plus doxorubicin and cyclophosphamide (FAC) as adjuvant chemotherapy for operable node-positive breast cancer and found that adjuvant chemotherapy with TAC significantly improves the rates of disease-free and overall survival among women with operable node-positive breast cancer [28].

**Table 3:** Enrichment Analysis of Breast Cancer Related Markers

PathwayID	PathwayName	N	P-value	AE	RE	MJI
hsa05212	Pancreatic cancer	72	3.74E-08	7	75.59	0.2819
hsa05213	Endometrial cancer	53	1.07E-07	6	88.02	0.2566
hsa05215	Prostate cancer	100	1.07E-07	7	54.43	0.2683
hsa05223	Non-small cell lung cancer	60	1.60E-07	6	77.75	0.25
hsa05218	Melanoma	72	3.55E-07	6	64.79	0.2417
hsa05200	Pathways in cancer	348	1.02E-06	9	20.11	0.3129
hsa05219	Bladder cancer	42	1.02E-06	5	92.56	0.2262
h_RacCycDPathway	Influence of Ras and Rho proteins on G1 to S Transition	26	6.66E-06	4	119.62	0.2103
hsa05214	Glioma	69	6.66E-06	5	56.34	0.2029
hsa05220	Chronic myeloid leukemia	74	7.91E-06	5	52.54	0.2005
hsa05166	HTLV-I infection	272	1.71E-05	7	20.01	0.2462
hsa05222	Small cell lung cancer	90	1.71E-05	5	43.2	0.1944
200124	E-cadherin signaling in the nascent adherens junction	39	2.10E-05	4	79.75	0.1846
200141	FOXM1 transcription factor network	41	2.37E-05	4	75.86	0.1821
200190	a6b1 and a6b4 Integrin signaling	46	3.40E-05	4	67.61	0.1768
hsa04110	Cell cycle	124	5.80E-05	5	31.35	0.1868
hsa05210	Colorectal cancer	62	8.23E-05	4	50.16	0.1656
200041	Signaling events mediated by Hepatocyte Growth Factor Receptor (c-Met)	80	1.67E-05	4	38.88	0.1583

**DiseaseID**	**DiseaseName**	**N**	**P-value**	**AE**	**RE**	**MJI**

MESH:D002528	Cerebellar Neoplasms	332	6.34E-07	9	32.39	0.3136
MESH:D020967	Myotonic Disorders	278	1.87E-05	7	30.09	0.2459
MESH:D042883	Choledocholithiasis	157	1.87E-05	6	45.67	0.2191
MESH:D002282	Adenocarcinoma, Bronchiolo-Alveolar	339	3.44E-05	7	24.68	0.2437
MESH:D009134	Muscular Atrophy, Spinal	1119	3.44E-05	11	11.75	0.3716
MESH:D016510	Corneal Neovascularization	669	3.44E-05	9	16.08	0.3067
MESH:D044483	Intestinal Polyposis	120	4.42E-05	5	49.79	0.1875
PA443756	Colonic Neoplasms	122	4.42E-05	5	48.98	0.1872
PA445062	Neoplasms	237	4.42E-05	6	30.25	0.2127
MESH:D007972	Leukoplakia, Oral	238	4.42E-05	6	30.13	0.2126
MESH:D003123	Colorectal Neoplasms, Hereditary Nonpolyposis	126	4.42E-05	5	47.42	0.1865
MESH:D046152	Gastrointestinal Stromal Tumors	148	8.01E-05	5	40.37	0.1836

**DrugID**	**DrugName**	**N**	**P-value**	**AE**	**RE**	**MJI**

PA451581	tamoxifen	74	1.83E-03	5	18.03	0.2124
PA131301952	gefitinib	39	1.83E-03	4	27.36	0.1941
PA152241907	lapatinib	14	1.83E-03	3	57.17	0.2143
PA449383	docetaxel	77	9.81E-03	4	13.86	0.1688
PA449509	estrogens	79	9.89E-03	4	13.51	0.1682

**OrganID**	**OrganName**	**N**	**P-value**	**AE**	**RE**	**MJI**

larynx	larynx	88	1.97E-2	2	25.45	0.2114

By the pathway analysis (p-value ≤ 1.69 × 10^-4^, *AE *≥ 3.03, *RE *≥ 20.01 and *MJI *≥ 0.158), we identified 18 associated pathways of which most are linked with cancer such as hsa05212 Pancreatic cancer, hsa05213 Endometrial cancer, hsa05215 Prostate cancer, hsa05223 Non-small cell lung cancer, hsa05218 Melanoma, hsa05219 Bladder cancer, hsa05200 Pathways in cancer, hsa05214 Glioma, hsa05220 Chronic myeloid leukemia, hsa05222 Small cell lung cancer, and hsa05210 Colorectal cancer (Table 3). We also discovered 107 diseases (p-value ≤ 1.59 × 10^-4^, *AE *≥ 4.35, *RE *≥ 6.31 and *MJI *≥ 0.17, Table 3, the top 12 diseases were shown due to space limitation). Most of them are linked with cancer such as MESH:D002528 Cerebellar Neoplasms, MESH:D016510 Corneal Neovascularization, MESH:D002282 Adenocarcinoma, Bronchiolo-Alveolar, MESH:D044483 Intestinal Polyposis, PA443756 Colonic Neoplasms, PA445062 Neoplasms, MESH:D003123 Colorectal Neoplasms, Hereditary Nonpolyposis, and MESH:D046152 Gastrointestinal Stromal Tumors.

By the inter-association, we found that the number 1 pathway (hsa05212, pancreatic cancer) we identified from the enrichment analysis is also highly associated with the pathway (hsa05200, pathways in cancer, p-value = 3.04 × 10^-66^, 46 orders of magnitude more significant than the pathway-pathway p-value threshold 2.13 × 10^-19^), disease (MESH:D046152 Gastrointestinal Stromal Tumors, p-value = 1.89 × 10^-32^, 25 orders of magnitude more significant than the pathway-disease p-value threshold 1.28 × 10^-6^), and drug (PA450191 lecithin, p-value = 4.55 × 10^-11^, 7 orders of magnitude more significant than the pathway-drug p-value threshold 5.73 × 10^-4^). Highly is measured by p-value. When the individual p-values are at least three orders of magnitude lower than current used p-value threshold, they are called "highly significant."

The pathway "hsa05200, pathways in cancer" and disease "MESH:D046152 Gastrointestinal Stromal Tumors" are already included in our previous enrichment analysis and were validated by the inter-association analysis. The drug PA450191 lecithin was filtered out in the enrichment analysis due to its insignificant measurement (p-value = 0.0472, AE = 2, RE = 9.04, MJI = 0.0884) and was discovered by the inter-association analysis as a previously undiscovered drug (p-value = 4.55 × 10^-11^, AE = 14, RE = 14.53, MJI = 0.2334). Similarly, the number 1 disease (MESH:D002528 Cerebellar Neoplasms) was found to be inter-associated with hsa05200 Pathways in cancer (validated, p-value = 6.86 × 10^-42^, AE = 79, RE = 9.39, MJI = 0.2536), MESH:D016410 Lymphoma, T-Cell, Cutaneous (previously undiscovered, p-value = 3.76 × 10^-100^, AE = 320, RE = 6.15, MJI = 0.5389), and PA449780 glutathione (previously undiscovered, p-value = 4.41 × 10^-18^, AE = 37, RE = 8.20, MJI = 0.3173); and the number 1 drug (PA451581 tamoxifen) was found to be inter-associated with 211859 Biological oxidations (previously undiscovered, p-value = 9.31 × 10^-25^, AE = 24, RE = 30.06, MJI = 0.2654), PA443560 Breast Neoplasms (previously undiscovered, p-value = 3.26 × 10^-50^, AE = 49, RE = 35.43, MJI = 0.4042), and PA449503 estradiol (previously undiscovered, p-value = 1.2 × 10^-21^, AE = 30, RE = 15.45, MJI = 0.3558).

Another dataset we used to assess the enrichment analysis is with the "self-validation" in Case Study 1. The self-validation makes the result of enrichment analysis more reliable and meaningful and consistent with the existing biological context. If a result of enrichment analysis can be validated by its subsequent inter-association analysis, it is also validated that the enrichment analysis and inter-association analysis are consistent and are both somewhat reliable.

### Assessment of inter-associated analysis

**Figure 5:**
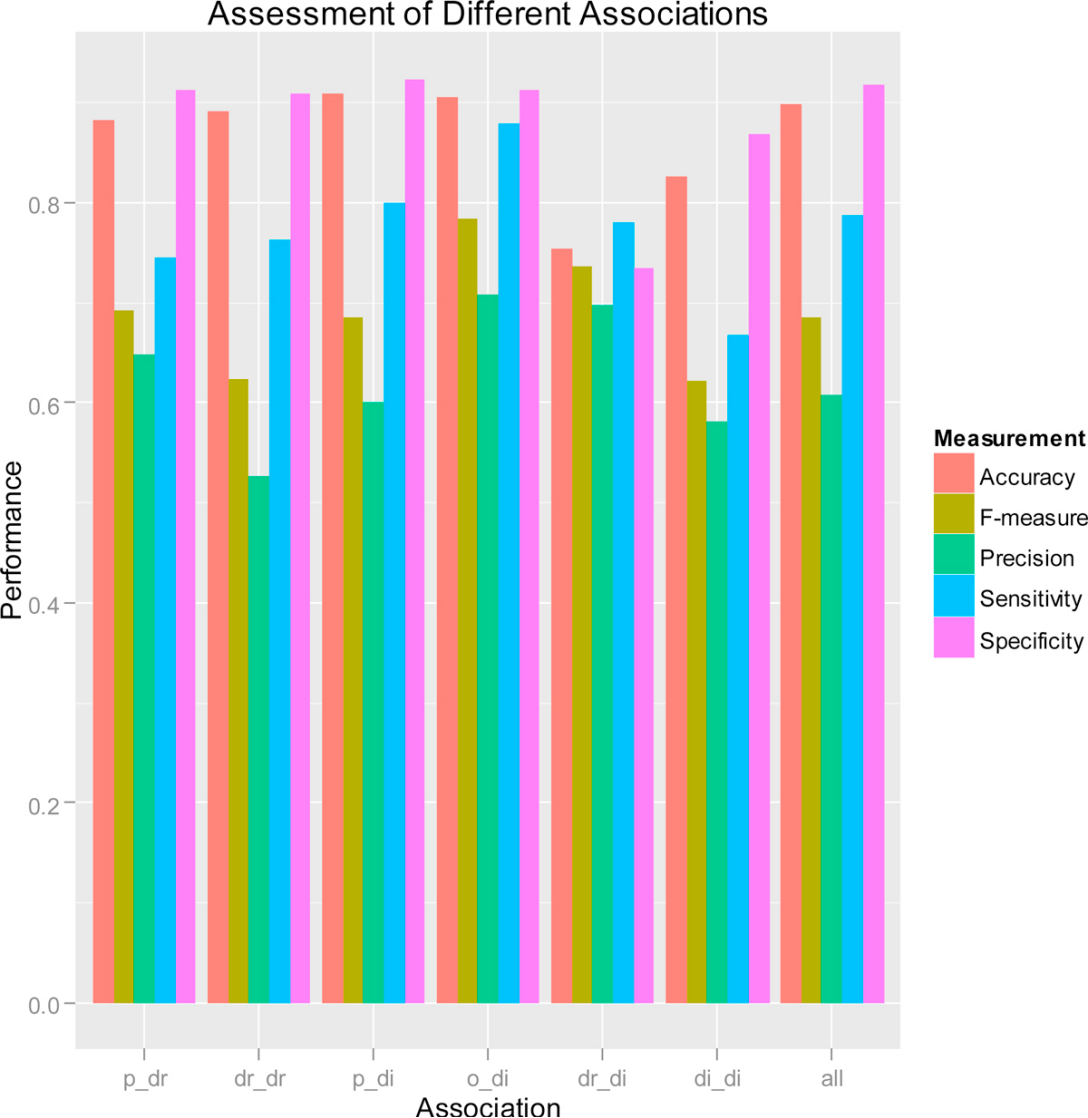
**Assessment of Different Associations**. The bar plot shows sensitivity, specificity, prediction, accuracy, and F_measure of pathway-drug, drug-drug, pathway-disease, organ-disease, drug-disease, disease-disease, and all associations as a whole.

We constructed a "gold standard" of 30161 inter-associations (247 Pathway-Drug; 274 Drug-Drug; 23659 Pathway-Disease; 405 Organ-Disease; 2826 Drug-Disease; 2750 Disease-Disease) from KEGG [5], CTD [17], PharmGKB [18], DrugBank [19], and Disease Ontology http://do-wiki.nubic.northwestern.edu/do-wiki/index.php/Main_Page[29]. We evaluated the performance of inter-association analysis method for the above six types of inter-associations (Figure 5). Pathway-Disease inter-associations have the highest specificity (92.4%), and Organ-Disease inter-associations have the highest sensitivity (87.9%) and F_measure (78.4%).

Compared to sensitivity, specificity and accuracy, the prediction rates are relatively low because the size of testing set are much larger than that of the "gold standard" set. When more "gold standards" of inter-associations become available in the future, the prediction rates and F_measure can be improved because the currently unpredicted pairs will be able to be predicted correctly. Figure 5 also gives a global evaluation for all 30161 inter-associations (Precision 60.73%, Accuracy 89.90%, Sensitivity 78.69%, Specificity 91.72%, F_measure 68.56%). Overall, the balanced F_measure (68.56%) shows our inter-association analysis method is reliable and can be used for further enrichment analysis.

## Case Studies

We show two case studies of increasing complexity and biological significance to achieve two goals: 1) to demonstrate the IPAD's ability to self-validate by using it to perform enrichment analysis and inter-association analysis on the 369 brain-specific markers, and 2) to demonstrate the ability of IPAD to identify previously undiscovered components by the enrichment analysis based on differentially expressed genes identified from a prostate cancer study.

### Case Study 1: Self-validation with inter-association analysis

**Figure 6:**
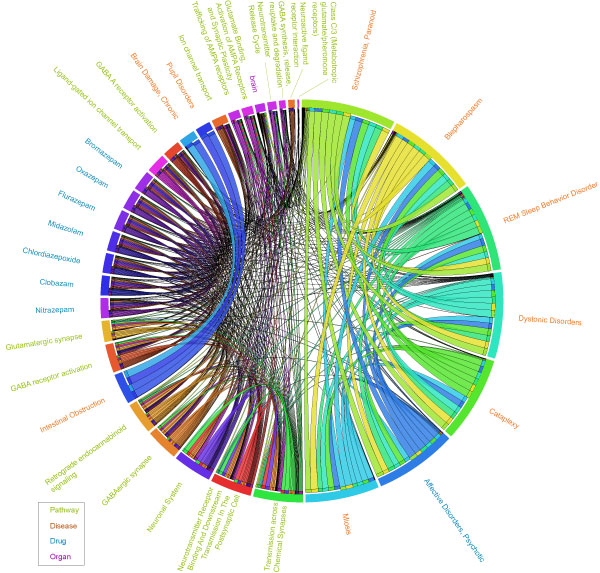
**A Circular View of the Inter-association Analysis of 369 Brain-Specific Markers**. The text colors for the four components: pathway, disease, drug, and organ are palegreen, Chocolate, royalblue, and magenta, respectively. Links with variable thickness represent the extent of association between two components which is 1 minus log10 of p-value. The direction of association (A->B) is represented by a ribbon's end touching A and its other end not touching B.

The highly associated relationships between pathway, disease, drug and organ can be used to validate the identified enriched pathway, disease, drug and organ candidates. The more dense and complex the inter-association between the four components, the more reliable and robust the identified candidates. In order to demonstrate the self-validation of IPAD, we first performed enrichment analysis on the 369 brain-specific markers we extracted from Homer [20] and then used the inter-association analysis in IPAD to validate the traditional enrichment analysis. We identified 16 enriched pathways (p-value ≤ 5.67 × 10^56^, *AE *≥ 4.86, *RE *≥ 7.42 and *MJI *≥ 0. 107), 92 enriched diseases (p-value ≤ 4.52 × 10^-7^, *AE *≥ 28.55, *RE *≥ 1.31 and *MJI *≥ 0.10), 7 enriched drugs (p-value ≤ 2.49 × 10^-7^, *AE *≥ 8.06, *RE *≥ 26.98 and *MJI *≥ 0.30), and 1 enriched organ (p-value ≤ 0.05, *AE *≥ 260, *RE *≥ 15.42 and *MJI *≥ 0.85) (Table 4, only 10 diseases are shown due to space limitation). All components were validated by the inter-association analysis except that only 88 out of 92 diseases were validated. Due to space limitation, we selected the top 10 diseases and other components to draw a circular view. The circular view of the 16 pathways, 7 drugs, 1 organ and top 10 diseases shows that all these 34 components are inter-associated with at least one other component (Figure 6). The table visualization was created by the tableviewer utility script, which is included in Circos [30]. We set the four text colors: palegreen, chocolate, royalblue, and magenta which stand for the four components: pathway, disease, drug, and organ, respectively. We transformed the extent of association between two components by using 1 minus log10 of p-value and set links with variable thickness representing the extent of inter-associations. The direction of association (A->B) is represented by a ribbon's end touching A and its other end not touching B.

**Table 4:** Enrichment Analysis of Brain-Specific Markers

Rank	PathwayID	PathwayName	N	Pvalue	AE	RE	MJI
1	112315	Transmission across Chemical Synapses	190	0	27	17.26	0.2117
2	112316	Neuronal System	283	0	32	13.74	0.2232
3	hsa04723	Retrograde endocannabinoid signaling	116	4.68E-14	18	18.85	0.1713
4	112314	Neurotransmitter Receptor Binding And Downstream Transmission In The Postsynaptic Cell	136	4.48E-13	18	16.08	0.1599
5	hsa04727	GABAergic synapse	98	5.48E-13	16	19.84	0.165
6	hsa04080	Neuroactive ligand-receptor interaction	401	4.42E-12	26	7.88	0.1678
7	977441	GABA A receptor activation	12	7.38E-09	7	70.87	0.3281
8	975298	Ligand-gated ion channel transport	25	1.41E-08	8	38.88	0.2017
9	977443	GABA receptor activation	53	1.30E-07	9	20.63	0.1318
10	hsa04724	Glutamatergic synapse	134	2.62E-07	12	10.88	0.1073
11	983712	Ion channel transport	61	3.20E-07	9	17.92	0.1206
12	420499	Class C/3 (Metabotropic glutamate/pheromone receptors)	15	5.19E-07	6	48.6	0.2313
13	888590	GABA synthesis, release, reuptake and degradation	19	1.52E-06	6	38.37	0.1891
14	399719	Trafficking of AMPA receptors	30	1.33E-05	6	24.3	0.1313
15	399721	Glutamate Binding, Activation of AMPA Receptors and Synaptic Plasticity	30	1.33E-05	6	24.3	0.1313
16	112310	Neurotransmitter Release Cycle	36	3.03E-05	6	20.25	0.1146

**Rank**	**DiseaseID**	**DiseaseName**	**N**	**Pvalue**	**AE**	**RE**	**MJI**

1	MESH:D001764	Blepharospasm	699	0	45	5.52	0.1398
2	MESH:D012563	Schizophrenia, Paranoid	649	3.18E-12	40	5.29	0.1265
3	MESH:D002385	Cataplexy	723	1.33E-11	41	4.86	0.1264
4	MESH:D020187	REM Sleep Behavior Disorder	506	1.44E-11	34	5.76	0.1149
5	MESH:D020821	Dystonic Disorders	837	4.52E-10	41	4.2	0.1226
6	MESH:D015877	Miosis	1000	1.94E-09	44	3.77	0.1273
7	MESH:D001925	Brain Damage, Chronic	1732	1.20E-08	59	2.92	0.1582
8	MESH:D000341	Affective Disorders, Psychotic	700	1.50E-08	34	4.17	0.1056
9	MESH:D007415	Intestinal Obstruction	1293	2.33E-08	48	3.18	0.1334
10	MESH:D011681	Pupil Disorders	1612	2.61E-08	55	2.93	0.1486

**Rank**	**DrugID**	**DrugName**	**N**	**Pvalue**	**AE**	**RE**	**MJI**

1	DB01595	Nitrazepam	20	2.92E-08	10	29.64	0.3294
2	DB00349	Clobazam	19	4.26E-08	9	28.08	0.3083
3	DB00475	Chlordiazepoxide	19	4.26E-08	9	28.08	0.3083
4	DB00683	Midazolam	19	4.26E-08	9	28.08	0.3083
5	DB00690	Flurazepam	19	4.26E-08	9	28.08	0.3083
6	DB00842	Oxazepam	19	4.26E-08	9	28.08	0.3083
7	DB01558	Bromazepam	19	4.26E-08	9	28.08	0.3083

**Rank**	**OrganID**	**OrganName**	**N**	**Pvalue**	**AE**	**RE**	**MJI**

1	brain	brain	363	0	260	15.42	0.8581

The 10 identified diseases: 1) MESH:D001764, Blepharospasm, 2) MESH:D012563, Schizophrenia, Paranoid, 3) MESH:D002385, Cataplexy, 4) MESH:D020187, REM Sleep Behavior Disorder, 5) MESH:D020821, Dystonic Disorders, 6) MESH:D015877, Miosis, 7) MESH:D001925, Brain Damage, Chronic, 8) MESH:D000341, Affective Disorders, Psychotic, 9) MESH:D007415, Intestinal Obstruction, and 10) MESH:D011681, Pupil Disorders, have on average 766 inter-associations between pathway, disease, drug and organ, which shows a strong association with those 369 brain-specific markers.

A blepharospasm is any abnormal contraction or twitch of the eyelid. There have been several important advances in understanding the brain mechanisms associated with blepharospasm. Baker et al. identified blinking-induced functional magnetic resonance imaging (fMRI) activation patterns in five benign essential blepharospasm (BEB) patients and five age-matched control subjects and concluded that the activations observed might represent a hyperactive cortical circuit linking visual cortex, limbic system, supplementary motor cortex, cerebellum, and supranuclear motor pathways innervating the periorbital muscles [31]. Antal et al. examined whether magnetic or electrical stimulation of the brain could improve the involuntary closure of the eyelids in patients with blepharospasm or Meige syndrome [32].

Schizophrenia is a brain disorder that affects the way a person acts, thinks, and sees the world. People with schizophrenia have an altered perception of reality, often a significant loss of contact with reality. Chen et al. utilized a multivariate approach to identify genomic risk components associated with brain function abnormalities in schizophrenia [33]. They first derived 5157 candidate single nucleotide polymorphisms (SNPs) from genome-wide array based on their possible connections with schizophrenia and further investigated for their associations with brain activations captured with functional magnetic resonance imaging (fMRI) during a sensorimotor task. Then, they identified 222 SNPs which showed significant difference between 92 schizophrenia patients and 116 healthy controls. Their further pathway analysis showed that the genes associated with the identified SNPs participated in four neurotransmitter pathways: GABA receptor signaling, dopamine receptor signaling, neuregulin signaling and glutamate receptor signaling. Their finding is consistent with our inter-association analysis from the 369 brain-specific markers.

Our 16 pathways identified by inter-association analysis using IPAD contains 1) Neurotransmitter Receptor Binding And Downstream Transmission In The Postsynaptic Cell, 2) Neuroactive ligand-receptor interaction, 3) GABAergic synapse, 4) GABA receptor activation, 5) Glutamate Binding, Activation of AMPA Receptors and Synaptic Plasticity, 6) Neurotransmitter Release Cycle, 7) GABA synthesis, release, reuptake and degradation, 8) Class C/3 (Metabotropic glutamate/pheromone receptors), and 9) GABA A receptor activation etc.

The other 7 diseases (except Intestinal Obstruction) also show strong links with brain, such as Cataplexy [34], REM Sleep Behavior Disorder [35], Dystonic Disorders [36], Miosis [37], Brain Damage [38], Chronic [39], Affective Disorders [40], Psychotic [41], and Pupil Disorders [42].

The 7 identified drugs: 1) DB00349, Clobazam, 2) DB00475, Chlordiazepoxide, 3) DB00683, Midazolam, 4) DB00690, Flurazepam, 5) DB00842, Oxazepam, 6) DB01558, Bromazepam, and 7) DB01595, Nitrazepam have on average 63 inter-associations between pathway, disease, drug and organ. They show strong links with brain, such as 1) Clobazam [43], 2) Chlordiazepoxide [44], 3) Midazolam [45], 4) Flurazepam [46], 5) Oxazepam [47], 6) Bromazepam [48], and 7) Nitrazepam [49].

In conclusion, this case study shows that the self-validation of IPAD is an innovation of traditional enrichment analysis and can be useful for validating any pathways, diseases, drugs or organs that users identify with their own data and methods.

### Case Study 2: Identification of previously undiscovered components by IPAD

RNA-seq is an emerging technology for surveying gene expression and transcriptome content by directly sequencing the mRNA molecules in a sample. RNA-seq can provide gene expression measurements and is regarded as an attractive approach to analyze a transcriptome in an unbiased and comprehensive manner. In this case study, we demonstrate the use of IPAD to identify previously undiscovered components by the enrichment analysis based on differentially expressed genes identified from the transcriptional profiling sequencing data [50]. The original purpose is to provide a general guide for analysis of gene expression and alternative splicing by deep sequencing. In the prostate cancer study, the prostate cancer cell line LNCap was treated with androgen/DHT. Mock-treated and androgen-stimulated LNCap cells were sequenced using the Illumina 1G Genome Analyzer. For the mock-treated cells, there were four lanes totaling ~10 million reads. For the DHT-treated cells, there were three lanes totaling ~7 million reads. All replicates were technical replicates. Samples labeled s1 through s4 are from mock-treated cells. Samples labeled s5, s6, and s8 are from DHT-treated cells. The read sequences are stored in FASTA files. The sequence IDs break down as follows: seq_(unique sequence id)_(number of times this sequence was seen in this lane). We first downloaded the publicly available transcriptional profiling sequencing data from the author's Web Site at http://yeolab.ucsd.edu/yeolab/Papers.html and computed the digital gene expression, next identified 278 differentially expressed genes in RNA-seq data from hormone treated prostate cancer cell line samples, then performed the enrichment analysis of the 278 genes with IPAD, and lastly carried out the inter-association analysis for these enriched components with IPAD.

In total, we identified 11 enriched pathways (p-value ≤ 5 × 10^-2^,*AE *≥ 3.45, *RE *≥ 1.95 and *MJI *≥ 0.040), 100 diseases(p-value ≤ 1.6 × 10^-3^, *AE *≥ 68.35, *RE *≥ 1.30 and *MJI *≥ 0.147), and 2 organs (p-value ≤ 1.9 × 10^-2^,*AE *≥ 4.38, *RE *≥ 5.45 and *MJI *≥ 0.080) for the 278 genes. And the further inter-association analysis of IPAD identified 10 pathways, 8 diseases, 2 drugs and 1 organs which are not previously discovered by the enrichment analysis of IPAD (Table 5).

**Table 5:** Identification of Previously Undiscovered Components by IPAD

PathwayID	PathwayName	p-value	AE	RE	MJI	C
1430728	Metabolism	4.32E-35	525	2.19	0.34	93
556833	Metabolism of lipids and lipoproteins	1.00E-13	175	2.25	0.27	91
453279	Mitotic G1-G1/S phases	2.41E-42	47	27.44	0.33	87
200137	AP-1 transcription factor network	2.36E-06	44	2.97	0.32	87
453279	Mitotic G1-G1/S phases	2.77E-09	78	2.73	0.30	87
200120	Direct p53 effectors	6.09E-07	70	2.4	0.27	85
69278	Cell Cycle, Mitotic	1.81E-14	166	2.37	0.28	82
1640170	Cell Cycle	1.51E-12	183	2.11	0.26	80
535734	Fatty acid, triacylglycerol, and ketone body metabolism	1.38E-07	81	2.35	0.26	79
71291	Metabolism of amino acids and derivatives	7.54E-15	120	2.9	0.33	76

DiseaseID	DiseaseName	p-value	AE	RE	MJI	C

MESH:D015228	Hypertriglyceridemia	1.60E-192	4573	2.09	0.77	79
MESH:D009468	Neuromuscular Diseases	4.34E-07	117	2.04	0.41	74
MESH:D009468	Neuromuscular Diseases	2.16E-293	4573	2.52	0.82	74
MESH:D052016	Mucositis	4.99E-199	4572	2.11	0.77	72
MESH:D002543	Cerebral Hemorrhage	7.21E-128	4572	1.8	0.73	72
MESH:D006463	Hemolytic-Uremic Syndrome	3.65E-215	4570	2.18	0.78	72
MESH:D020246	Venous Thrombosis	6.59E-106	4573	1.71	0.72	70
MESH:D013923	Thromboembolism	1.78E-123	4573	1.79	0.73	70

DrugID	DrugName	p-value	AE	RE	MJI	C

PA449383	Docetaxel	3.08E-02	52	1.89	0.36	24
PA449780	Glutathione	1.74E-10	20	10.48	0.24	13
PA131301952	Gefitinib	8.52E-17	29.88	10.84	0.395	8
PA451283	Rosiglitazone	7.73E-22	41.4	10.58	0.386	5
PA448803	Carboplatin	2.57E-15	27	11.36	0.397	5

OrganID	OrganName	p-value	AE	RE	MJI	C

liver	Liver	1.82E-17	179	2.72	0.40	84

We found that some of these components that were previously undiscovered but identified by inter-association analysis still showed strong association with prostate cancer. For example, previous studies reported that the top 5 drugs we identified with inter-association analysis: docetaxel, glutathione, gefitinib, rosiglitazone, and carboplatin were all associated with prostate cancer. Docetaxel is a drug used in men whose prostate cancer no longer responds to hormone therapy. Tannock et al. compared docetaxel plus prednisone in men with advanced, hormone-refractory prostate cancer with mitoxantrone plus prednisone. They found that treatment with docetaxel every three weeks led to superior survival and improved rates of response in terms of pain, serum PSA level, and quality of life, as compared with mitoxantrone plus prednisone, when given with prednisone [51]. The deficiency in the glutathione enzyme system has been proposed to increase the likelihood of developing both an enlarged prostate and prostate cancer. Nelson discovered a genetic defect in prostate cancer cell prevents the body from producing glutathione S-transferase (GST), an enzyme needed by the liver to detoxify harmful chemicals [52]. The function of a particular glutathione enzyme glutathione-S-transferase-pi-i (GSTP1) is almost universally lost in both cancerous and pre-cancerous prostate cells. The inactivation of this glutathione enzyme is an early event in the development of prostate cancer. Many studies have linked the loss of GSTP 1 to malignant transformation of prostatic tissues [52].

One study found that gefitinib and bicalutamide showed synergistic effects in primary cultures of prostate cancer derived from androgen-dependent naive patients [53]. Another study discovered that gefitinib-trastuzumab combination showed promising clinical activity in hormone refractory prostate cancer [54]. Smith et al. assessed the biological activity of rosiglitazone, a peroxisome proliferator-activated receptor gamma agonist that has been approved to treat type 2 diabetes, in men with recurrent prostate carcinoma using change in prostate specific antigen (PSA) doubling time (PSADT) as the primary outcome variable and concluded that Rosiglitazone did not increase PSADT or prolong the time to disease progression more than placebo in men with a rising PSA level after radical prostatectomy and/or radiation therapy [55]. But Rosiglitazone was found to suppress human lung carcinoma cell growth through PPARγ-dependent and PPARγ-independent signal pathways [56]. The number 3 drug, Carboplatin is a chemotherapy agent used for treatment of many types of cancer. Some studies examined the efficacy of carboplatin as a second line chemotherapy agent (after the failure of taxotere) as well as along with taxotere therapy for men with advanced prostate cancer [57,58]. A phase II study assessed the outcome and predictive factors for prognosis and toxicity following intermittent chemotherapy with docetaxel, estramustine phosphate, and carboplatin (DEC) in patients with castrate resistant prostate cancer (CRPC) and found that combination chemotherapy with DEC has a potential effect on CRPC with acceptable toxicity [59]. Jeske et al. conducted a retrospective, bi-institutional review of patients with advanced CRPC treated with carboplatin plus paclitaxel after docetaxel and concluded that Carboplatin/paclitaxel chemotherapy following docetaxel in metastatic CRPC is well tolerated with favorable PSA response rates and survival and the combination is a viable option after progression on docetaxel-based therapy [60].

This case study shows that compared to traditional enrichment analysis, the IPAD's inter-association analysis can be more powerful and useful in identification of previously undiscovered pathways, diseases, drugs or organ specification.

## Conclusion

We developed IPAD as an integrated database system to analyze, identify, and validate pathway, disease, drug, organ specificity and their inter-associations. IPAD integrates many different types of pathway, disease, drug and organ-specificity information: pathway gene relationship from the BioCarta [4], KEGG [5], NCI-Nature curated [6], and Reactome [7] database; disease gene relationship from the CTD [17] and PharmGKB [18] database; drug gene relationship from the DrugBank [19] and PharmGKB [18] database; and organ-specific genes/proteins from the HOMER [20] databases.

Enriched pathways, diseases, drugs, organs and their inter-associations can be searched, displayed, and downloaded from our online user interface. The current IPAD database can help users address a wide range of pathway related, disease related, drug related and organ specificity related questions in human disease studies. We also developed a statistical method for similarity measurement and statistics and described two criteria for setting the threshold parameters, which can be extended to other enrichment applications. Lastly, our database was evaluated by comparison to other known databases, a constructed "gold standard" of 30161 known associations, and two case studies.

## Discussion

In this paper, we have demonstrated that IPAD can be used to discover, analyze, and validate pathway, disease, drug, and organ specificity from experimental data. We illustrated the features of IPAD by testing the inter-association between breast cancer markers related pathway, disease, drug and organ. In Case Study 1, we demonstrated the IPAD's ability to self-validate by using it to perform enrichment analysis and inter-association analysis on the 369 brain-specific markers. In Case Study 2, we further demonstrated the ability of IPAD to identify previously undiscovered components by the enrichment analysis based on differentially expressed genes identified from a prostate cancer study.

Selecting the appropriate statistical parameters for enrichment analysis and inter-association analysis is important. We presented a novel algorithm to measure relationships among the annotation terms based on p-value, Absolute Expression Value (*AE*), Relative Expression Value (*RE*) and Mean Jaccard Index (*MJI*). We also described the two criteria for setting the threshold parameters: 1) p-value below the 5% quantile and 2) 1 sigma lower control limits for *AE*, *RE *and *MJI*. However, defining each threshold parameter and implementing them effectively can be still challenging. Because the gene list size affects the enrichment score and the sizes of four types of component are largely different (Table 1, 11663 molecules in 1956 Pathways, 17925 molecules in 6704 diseases, 3735 molecules in 5615 drugs, and 5599 molecules in 52 organs).

In our website we provide all results for users to cut off according to the specificity of their input data. The number of enriched component sets depends on the structure of the data and the problem space. If no enriched component sets or a very large number of enriched component sets pass the thresholds, users first check whether too few or too many genes are loaded. If there are no such issues, users can tighten up the thresholds for too many significant component sets and relax them for no significant component sets.

In this paper, we introduced organ-pathway, organ-disease, organ-drug, organ-organ inter-associations for the first time. An organ actually means organ specificity in the paper. An organ is a group of tissues that perform a specific function or group of functions. Organ specificity is referred as the specificity of level of expression of a gene or protein in a certain type of organ. Identification of the association of organ-gene, organ-pathway, organ-disease, organ-drug, and organ-organ can be helpful in the discovery potentially therapeutic genes related to specific organs, measuring and understanding the function and characteristics of cells and tissues in an organ from the perspective of cooperative network, disease diagnosis, and drug target, indicating important clues about gene function, network signaling, disease treatment and drug target, and monitoring organ integrity both during preclinical toxicological assessment and clinical safety testing of investigational drugs.

## Methods

### Data sources

We show an overview of the data integration process in Figure 1. Pathway data in IPAD were collected from the four most commonly used sources, i.e., BioCarta [4], KEGG [5], NCI-Nature curated [6], and Reactome [7].

The BioCarta [4] includes expert-curated interactive graphic models of many pathways from diverse fields like apoptosis, cell cycle, cell signaling, development, immunology, neuroscience, adhesion, and metabolism. BioCarta data from June 2004 was imported from its website.

The KEGG [5] pathway is a collection of manually drawn pathway maps containing the knowledge on the molecular interaction and reaction networks in Metabolism, Genetic Information Processing, Environmental Information Processing, Cellular Processes, Organismal Systems, Human Diseases, and Drug Development. The KEGG data was downloaded from its ftp site.

The NCI-Nature curated [6] are created by Nature Publishing Group editors and reviewed by experts in the field. Biomolecules are annotated with UniProt protein identifiers and relevant post-translational modifications. Interactions are annotated with evidence codes and references. The NCI-Nature curated data was downloaded from its website.

Reactome [7] is an expert-authored, peer-reviewed knowledgebase of human reactions and pathways that provides infrastructure for computation and data mining across the biologic reaction network. Human pathways from Reactome were downloaded from its website.

Disease data in IPAD was downloaded from two different sources: CTD [17] and PharmGKB [18]. The Comparative Toxicogenomics Database CTD [17] is a public website and research tool that curates scientific data describing relationships between chemicals, genes, and human diseases. The Pharmacogenetics Knowledge Base (PharmGKB) [18] is curate knowledgebase about the impact of genetic variation on drug response with focus on clinical interpretation of variants associated with drug response, drug dosing guidelines and genetic tests, drug-centered pathways, important PGx gene summaries, and relationships among genes, drugs and diseases.

Drug data in IPAD were downloaded from two different sources, DrugBank [19] and PharmGKB [18]. The DrugBank database [19] is a unique bioinformatics and cheminformatics resource that combines detailed drug (i.e. chemical, pharmacological and pharmaceutical) data with comprehensive drug target (i.e. sequence, structure, and pathway) information.

The organ specificity in IPAD was downloaded from HOMER [20]. HOMER [20] is an integrated Human Organ-specific Molecular Electronic Repository, defining human organ-specific genes/proteins and covering about 22,598 proteins, 52 organs, and 4,290 diseases integrated and filtered from organ-specific proteins/genes and disease databases like dbEST [61], TiSGeD [62], HPA [63], CTD [17], and Disease Ontology [29].

We used PERL to parse the text data we downloaded and a light-weight implementation of the Document Object Model interface in Python 2.7.l [64], xml.dom.minidom to parse the XML format data.

### Similarity measure for the inter-association analysis

The Jaccard Index measures similarity between pathways, diseases, drugs and organs, and is defined as the size of the intersection divided by the size of the union of the component sets. The component similarity measure can be defined as the extent of overlaps, e.g., common number of genes/proteins, shared between two different components [65]. In IPAD, we have four types of components: pathway, disease, drug and organ.

The component-component similarity score JI*_i,j _*is defined as Jaccard Index,

$$JI_{i,j}=\frac{\left|P_{i}\cap P_{j}\right|}{\left|P_{i}\cup P_{j}\right|}i=1...N,j=1...M,$$

where, *N*, *M *denotes total number of components. *P_i _*and *P_j _*denote two different components, *P_i _*and *P_j _*can be the same or different type, while |*P_i_*| and |*P_j_*| are the numbers of molecules in these two components. Their intersection *P_i _*∩*P_j _*is the set of all molecules that appear in both *P_i _*and *P_j_*, while their union *P_i _*∪*P_j _*is the set of all molecules either appearing in the *P_i _*or in the *P_j_*. Duplicates are eliminated in the intersection set and union set.

Similarly, we define the left component-component similarity score LJI*_i,j _*as Left Jaccard Index,

$$LJI_{i,j}=\frac{\left|P_{i}\cap P_{j}\right|}{min\left(\left|P_{i}|,|P_{j}\right|\right)}i=1...N,j=1...M,$$

the right component-component similarity score RJI*_i,j _*as Right Jaccard Index,

$$RJI_{i,j}=\frac{\left|P_{i}\cap P_{j}\right|}{max\left(\left|P_{i}|,|P_{j}\right|\right)}i=1...N,j=1...M,$$

and the mean component-component similarity score MJI*_i,j _*as Mean Jaccard Index,

$$MJI_{i,j}=\frac{LJI_{i,j}+RJI_{i,j}}{2}i=1...N,j=1...M.$$

With the equations above, we can calculate similarity scores (Jaccard Index, Left Jaccard Index, Right Jaccard Index, and Mean Jaccard Index) for pathway-pathway, disease-disease, drug-drug, organ-organ, pathway-disease, pathway-drug, pathway-organ, disease-drug, disease-organ, and drug-organ associations.

### Statistics for the inter-association analysis

In addition to similarity scores, we developed a statistic model based on Fisher Exact test [66,67] and number of genes involved in a component for systematic enrichment analysis. When members of two independent groups can fall into one of two mutually exclusive categories, Fisher Exact test [66,67] is used to determine whether the proportions of those falling into each category differs by group. In IPAD enrichment system, Fisher Exact test is adopted to measure the gene-enrichment in annotation terms and the enrichment between components. Given *p *to be the probability of success in a Bernoulli trial where one gene in component *i *falls in component *j*, the probability of *x *successes is

$$P\left(x\right)=C_{L}^{x}p^{x}{\left(1-p\right)}^{L-x},$$

Where *L *is the total number of genes in component *i*, *M *is the total number of genes in component *j*, *N *is the total number of genes in the type of component, *p *= *M*/*N*, *x *is the number of genes corresponding to component *i *in component *j*, and $C_{L}^{x}$ is the number of possible combinations of *x *genes from a set of *L *genes.

The p-value for component *i *in component *j *is the probability of obtaining a test statistic at least as extreme as the one observed, given that the null hypothesis that there is no enrichment between component *i *and component *j *is true, and calculated according to the following formula

$$Pvalve=\sum_{x}^{M}P\left(x\right).$$

To prevent multiple testing problem from happening, IPAD adjust the p-value by Benjamini & Hochberg method [68].

The absolute enrichment value (*AE*) of component *i *in component *j *is defined as *x*, the number of genes corresponding to component *i *in component *j*. The expected enrichment value (*EE*) of component *i *in component *j *is defined as the expected number of genes of component *i *in component *j *under the null hypothesis that the component *i *and component *j *are independent of each other.

$$EE=L\cdot \frac{M}{N}.$$

The relative enrichment value (*RE*) of component *i *in component *j *is defined as *AE*/*EE*.

We define inter-associations as enriched ones if they satisfy the thresholds in table 6 (i.e. for Pathway-Pathway association: p-value ≤ 2.13^-19^, *RE *≥ 3.131, *AE *≥ 9 and *MJI *≥ 0.328; and so on). We determine the parameters based on the following two criteria: 1) Associations with p-value below the 5% quantile are chosen as enriched associations based on the p-value distribution of inter-association in the Figure 2 and the comparison of the five quantile thresholds in Table 7. 2) 1-sigma limits (1-standard error) are used to set the lower control limits for *AE*, *RE *and *MJI*. There are no upper control limits for *AE*, *RE *and *MJI*. *AE*, *RE *and *MJI *must be greater than or equal to one standard deviation from their means. Associations falling below the lower control limits are considered to be not stably enriched.

**Table 6:** Thresholds for Inter-association Analysis in IPAD

Typea	Typeb	p-value≤	AE≥	RE≥	MJI≥
pathway	Pathway	2.13E-19	9.000	3.131	0.328
Pathway	Disease	1.28E-06	3.000	1.268	0.127
Pathway	Disease	5.73E-04	2.168	2.133	0.193
Pathway	Organ	5.00E-02	1.132	1.970	0.109
Disease	Pathway	1.02E-05	3.000	1.254	0.140
Disease	Disease	4.19E-72	73.538	1.370	0.393
Disease	Drug	5.00E-02	2.000	1.422	0.171
Disease	Organ	5.00E-02	1.000	1.313	0.121
Drug	Pathway	1.60E-05	2.666	2.468	0.141
Drug	Disease	6.51E-03	1.000	1.358	0.133
Drug	Drug	1.59E-05	3.000	3.391	0.333
Drug	Organ	5.00E-02	2.678	3.856	0.201
Organ	Pathway	4.01E-02	1.000	2.018	0.056
Organ	Disease	6.27E-03	2.000	1.384	0.085
Organ	Drug	5.00E-02	2.206	3.093	0.155
Organ	Organ	5.00E-02	7.000	4.279	0.095

**Table 7:** A Comparison of the Five Quantile Thresholds

	# Associations In Pathway	#Associations In Disease	#Associations In Drug	#Associations In Organ	#total	F_measure
Quantile 3%	111374	594647	119170	3627	828818	60.75%
Quantile 4%	148455	786699	124467	4471	1064092	68.58%
Quantile 5%	185474	984366	130029	4471	1304340	68.56%
Quantile 6%	222175	1176166	135915	4471	1538727	68.18%
Quantile 7%	259592	1367923	143947	4471	1775933	66.92%

P-value below the 5% quantile performs better than other p-value thresholds with a balanced F_measure and an appropriate total number of inter-associations (Table 7). First, the threshold (p-value ≤ Quantile 3%) is too strict. It filters out about half of the inter-associations that are identified by the threshold (p-value ≤ Quantile 7%). Secondly, the thresholds (p-value ≤ Quantile 6%) and (p-value ≤ Quantile 7%) cannot perform better in F_measure than the threshold (p-value ≤ Quantile 5%). Finally, we choose (p-value ≤ Quantile 5%) as the best threshold because we can identify 23% more inter-associations with (p-value ≤ Quantile 5%) than with (p-value ≤ Quantile 4%), although the F_measure of the threshold (p-value ≤ Quantile 4%) is a little bit higher than that of the threshold (p-value ≤ Quantile 5%).

Further comparison between four sigma thresholds (Table 8) shows that 1-sigma threshold to set the lower control limits for *AE*, *RE *and *MJI *can have the better prediction performance than other sigma thresholds.

**Table 8:** A Comparison of the Four Sigma Thresholds

	# Associations In Pathway	#Associations In Disease	#Associations In Drug	#Associations In Organ	#total	F_measure
0.5 Sigma	117535	644957	58579	2222	823293	60.58%
1 Sigma	185474	984366	130029	4471	1304340	68.56%
2 Sigma	223000	1215652	156337	5329	1600318	67.78%
3 Sigma	223000	1215652	156337	5329	1600318	67.78%

### Similarity measure and statistics for the enrichment analysis

If a user's gene list is treated as a component, then the similarity measures and the statistics for genes-pathway, genes-disease, genes-drug and genes-organ can be similarly computed with the equations in the sections: "Similarity Measure for the Inter-association Analysis" and "Statistics for the Inter-association Analysis".

### Performance measurements

A "gold standard" of 30161 inter-associations (247 Pathway-Drug; 274 Drug-Drug; 23659 Pathway-Disease; 405 Organ-Disease; 2826 Drug-Disease; 2750 Disease-Disease) was constructed from KEGG [5], CTD [17], PharmGKB [18], DrugBank [19], and Disease Ontology [29] for *performance evaluation purpose *only. The following measurements were involved in our evaluation. (1) Sensitivity (also called recall) is the proportion of actual positive pairs which are correctly identified; (2) Specificity measures the proportion of negative pairs which are correctly identified; (3) Precision is the probability of correct positive prediction; (4) F_measure is the harmonic mean of precision and recall; (5) Accuracy is the proportion of correctly predicted pairs.

$$\begin{array}{c} Sensitivity=\frac{TP}{TP+FN}\\ Specificity=\frac{TN}{TN+FP}\\ Precision=\frac{TP}{TP+FP}\\ F\text{\_}measure=\frac{2*Precision*Sensitivity}{Precision+Sensitivity}\\ Accuracy=\frac{TP+TN}{TP+TN+FP+FN} \end{array}$$

### Online IPAD server design

The online version of IPAD database is a typical 3-tier web application [69], with an SQL Server2008R2 database at the backend database service layer, Apache/PHP server scripts to the middleware application web server layer, and CSS-driven web pages presented on the browser.

**Figure 7:**
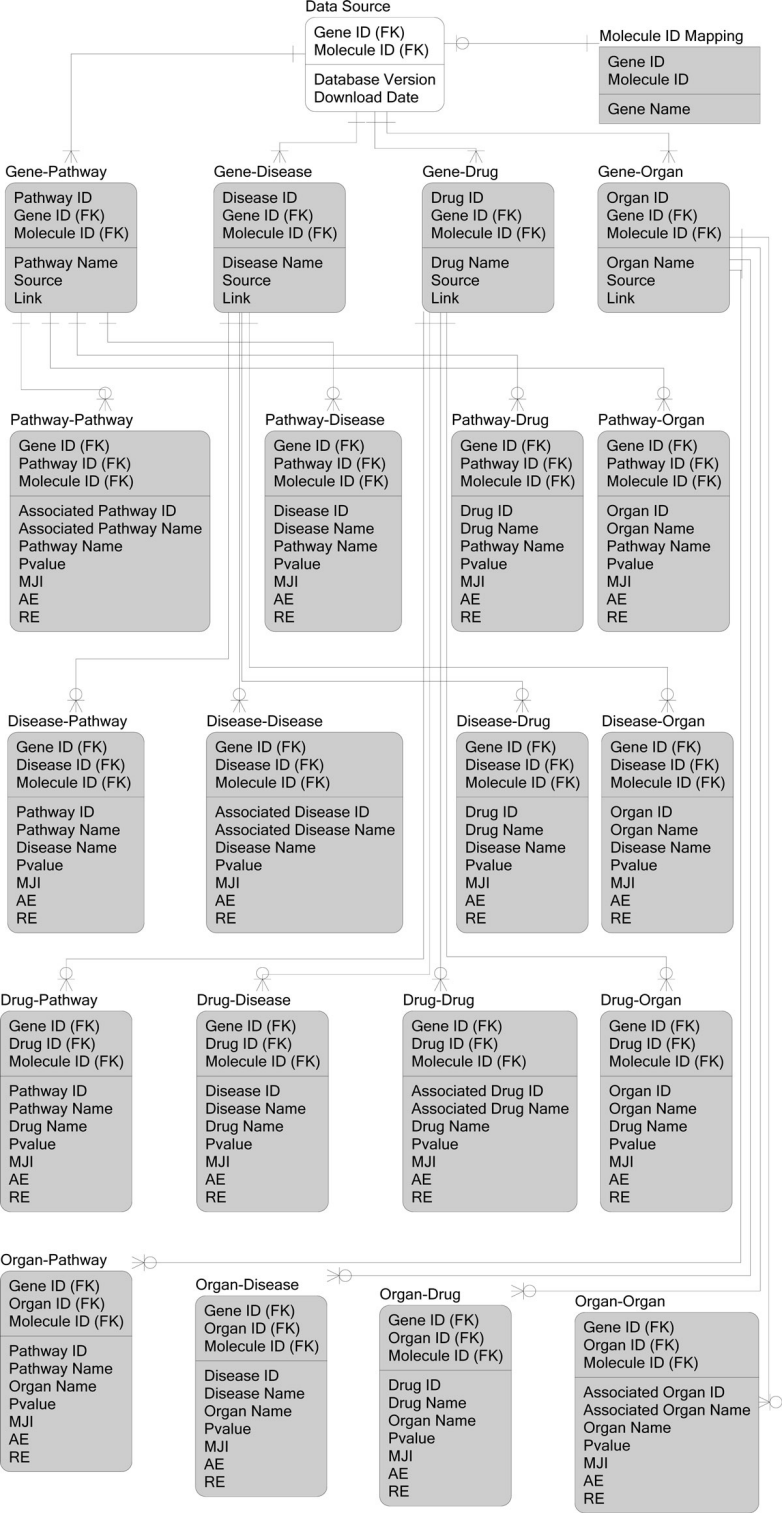
**Relational Metadata Model**. The datasets derived by the data generation pipeline are filled in gray.

The result tables derived from the data generation steps were imported into the SQL Server2008R2 database (Figure 7). The pathway-gene, disease-gene, drug-gene, organ-gene, pathway-disease, pathway-drug, pathway-organ, disease-drug, organ-disease, organ-drug tables enable users to query the database with different IDs.

## Competing interests

The authors declare that they have no competing interests.

## Authors' contributions

RD conceived the initial work and designed the method for the database construction. FZ generated the datasets, developed the statistics method, the database backend and the web-based interface, and performed the statistical analyses of the case studies. All authors are involved in the drafting and revisions of the manuscript.
